# Teaching communication and patient education in nursing: A case study grounded in self-regulated learning and motivation theory

**DOI:** 10.1016/j.pecinn.2026.100467

**Published:** 2026-03-09

**Authors:** Brynja Ingadóttir, Ásta Bryndís Schram, Helga Sif Friðjónsdóttir, Jennifer A. Wentzel

**Affiliations:** a University of Iceland, School of Health Sciences, Vatnsmýrarvegur 16, 101 Reykjavik, Iceland; b Landspitali – the National University Hospital of Iceland, Skaftahlid 24, 105 Reykjavik, Iceland

**Keywords:** Communication, Curriculum, Motivation, Nurse education, Nursing, Patient education, Self-regulation

## Abstract

**Objectives:**

To describe the preparation, testing, and evaluation of a new undergraduate course on communication and patient education using student feedback, self-regulation theory, and motivational theory.

**Methods:**

An exploratory case study design with data collected between 2018 and 2023 in an Icelandic university. Data sources were teachers' meeting memos and notes on the course design and development, nursing students' diaries on the teaching methods, and a survey where students evaluated their own motivation (using the MUSIC® Model of Motivation Inventory), their self-regulation (using the Student Perceptions of Classroom Knowledge-Building survey) and the course's teaching methods and environment.

**Results:**

The design of the course was based on Bandura's social cognitive theory, self-regulation theory and motivation theory. Students were found to be motivated, but struggled with self-regulation, particularly regarding the new teaching methods. The faculty learned that new modalities needed to be introduced slowly and interwoven through the whole nursing curriculum to be most impactful. Students needed to be taught how to use the methods and develop a level of comfort before they could be fully effective. While introducing active learning methods was challenging for both teachers and students, adding diverse, evidence-based teaching methods into the curriculum supports the development of complex competencies, like communication and patient education.

**Conclusions:**

Teaching methods and content that support students' continuous and active involvement is challenging for both teachers and students. They require thorough preparation and training along with supporting infrastructure from the university.

**Innovation:**

Incorporating diverse teaching modalities to foster the development of the interpersonal role of nursing are described. While active, experiential teaching and learning methods are encouraged in nursing education, it is crucial to understand how to support students who are new to these methods, so they can use them effectively.

## Highlights

• Gradually adding experiential learning with scaffolding helps support students’ motivation and self‑regulation.
• Student feedback helps guide the complex shift from a lecture‑based curriculum to one grounded in experiential learning practices.
• Caring teachers can help motivate students, even if their interest in the subject is low.

## 1 Introduction

The role of nurses is comprehensive and includes enhancing health literacy, preventing illness, health promotion, facilitating recovery and adaptation, and protecting patient safety [1]. Nurses serve as the primary link between patients and the interprofessional healthcare team, making their communication skills essential for coordinating care effectively. Among the professional values of nurses are respect, empathy and compassion [2] which can and should be developed in academic education. The growing body of evidence in compassionomics, the study of economic and clinical benefits of compassionate care, underscores that empathy and compassion are essential cornerstones of caring for patients. Research indicates that compassionate care can lead to improved patient satisfaction, better adherence to medical recommendations and faster recovery, contributing to lower healthcare costs [3].

Patient education also plays a significant role in improving care quality by empowering patients with knowledge about their health. Patient education can improve physiological, physical and psychological patient outcomes and general function [4]. Effective patient education requires nurses to not only convey information but also to tailor their communication to the patient's level of understanding and cultural background [5]. Here, patient centredness and patient engagement are core concepts [6]. However, several barriers have been identified that hinder nurses from providing patient education, the most reported one being lack of knowledge and skills [7].

Training nursing students in caring communication and patient education helps them to understand the significant impact of empathy on patient experiences and outcomes and produces future nurses who are not only clinically competent but will be able to provide care based on empathy and emotional intelligence [8]. Thus, the integration of comprehensive communication and patient education training in undergraduate nursing programs is essential for preparing future nurses to deliver safe, high-quality, and compassionate care that meets patients' needs and the complex demands of modern healthcare. However, communication competencies are generally not established in nursing education [9].

In Europe, the Bologna declaration of 1999 led to the development of the European Higher Education Area (EHEA) in 2010 to ensure mobility and recognition of diplomas [10]. Iceland has been a full member of the Bologna Process since the original declaration in 1999 [11]. Though the degrees and credits can transfer, no specific competencies are required by the EHEA. This gap drove the creation of the Health Professions Core Communication Curriculum (HPCCC) for undergraduate education in Europe [12]. This curriculum established clear curriculum objectives for all health professions but has not yet been studied in the context of nursing [13].

The primary communication framework in health professions continues to be the Calgary-Cambridge Guide which was developed for physician-patient interactions [13]. However, it does not translate well to the less-structured communication style of nursing [13]. Nurses rarely have sit-down, formal conversations with patients and family members like physicians, and therefore need a framework that works in practice [14]. Kerr et al. [14] suggested setting nursing communication skills within the praxis of nursing, which requires a new communication framework.

In a study of Bachelor of Science in Nursing (BSN) programs in the U.S., Wittenberg et al. [9] reported that only 24% of nursing programs include a required communication class. While 68% of programs had some class content on communication, many didn't include that training until the third or fourth year of study. A study soliciting student feedback on a communication curriculum found that students would prefer communication training to start as early as possible [15]. That study also found that students preferred real-life examples, patient simulations, and more concrete language of what to say to patients [15].

Gutierrez-Puertas et al. [8] found in their systematic review that simulation, including standardized patients, role-playing, or high-fidelity patient simulators, are effective methods for training communication in nursing students. They also recommended communication training begin in year one and continue throughout the program [8]. While the literature includes a variety of recommendations on potential educational interventions, nursing education needs behavioural, objective outcome measures supported by a standardized curriculum [14].

The university BSN program started in Iceland in 1973 when the diploma program was abolished. The BSN is a four-year program, available at either the University of Iceland in Reykjavik, or the University of Akureyri. At the University of Iceland, the number of enrolled students has gradually increased, from 80 in 2017 to 127 in 2023. The nursing curriculum was last revised in 2017, when a gap in communication education was noted. A decision was made to develop a specialised communication course, highlighting patient education. Until then, the subject of communication had been touched on in several courses in the curriculum but without a holistic, comprehensive approach. This course would be a requirement in the curriculum and occur in the fall of the students' second year of study. The HPCCC [12] and the Health Care Education Association (HCEA) Patient Education Guidelines for Health Care Professionals [6] were used to develop and refine the competencies and educational modalities.

The purpose of this exploratory case study is to describe the design and development of a feasible, evidence-based course in communication and education using student feedback, self-regulation theory, motivation theory and experiential learning methods. The focus is on obtaining an empirically based understanding of the course's structure and the teaching context. While there is a lot of evidence supporting experiential learning, the realities of restructuring a lecture-based curriculum are much more complex. As there is a very limited literature published on how to design such a course, this paper is presented as a case study to help provide context and enumerate the real-life challenges of implementing experiential learning in a large group university setting [16].

More specifically we aimed to answer the following research questions:

1. How was the traditional, lecture-based curriculum restructured into an active, experiential learning format for a large cohort of students?
2. How do students perceive their motivation and self-regulation after completing the new communication and patient education course?
3. How do students perceive the diverse evidence-based teaching methods chosen for the course and how can their feedback be used to support the development of such a course?
4. What lessons were learned during the development, implementation and evaluation stages that can inform future curriculum reforms?

## 2 Course development

The course development consisted of choosing a theoretical framework, designing, testing and evaluating the course using student feedback and teachers' experience.

### 2.1 Theoretical framework

The development of the course was guided by Bandura's social cognitive theory [17] and Kolb's experiential learning theory which emphasizes learning by doing [18]. Bandura's theory emphasizes that learning takes place in a social context with a dynamic and reciprocal interaction between the student, the learning environment, and actions. If students are to interact in a dynamic, reciprocal way with each other in practicing patient communication, they need to be motivated and engaged. In addition, they need to understand the purpose of the activity and be able to self-regulate, a practice that includes the important phase of self-reflection.

Student motivation is strongly related to learning and achievement [19]. Highly motivated students engage in their learning. Students´ perceptions of the learning environment can affect their motivation and engagement [20], [21]. Thus, it is important to design a course based on established motivational strategies. The MUSIC® Model of Motivation was used as the conceptual model for the design of the course [22], [23]. It is based on several theories and years of research on motivation e.g., Deci and Ryan [24], Hidi and Renninger [25], Pintrich et al. [26] and Wigfield and Eccles [27]. The name is an acronym which describes five factors that influence motivation and engagement when present in the teaching environment. These factors are eMpowerment (M), Usefulness (U), Success (S), Interest (I), and Caring (C).

Students are more motivated, self-directed, empowered and engaged when they feel that they have some choices and some flexibility in their studies (M), find their studies useful (U), believe that they can be successful in their studies (S), are interested in the topic (I), and finally, when they perceive that the teachers care how well they do in the course (C). The design of the course was therefore guided by these motivational strategies and students' perceptions of the motivational factors were used to inform the development of the course in order to increase their motivation further. This is particularly important in a course where developing skills for communication and patient education is the goal.

In a university setting, where successful learning of practical training skills is very much based on engagement and participation, students need to be self-regulated. Self-regulation has been defined as “the process whereby students activate and sustain cognitions, behaviours, and affects that are systematically oriented toward attainment of their goals” [19] (p.158). Self-regulation is key to successful learning in higher education. Consequently, it is important to glean students´ success in this regard.

### 2.2 Designing the course

The core concepts in nursing guided the course development, i.e. caring, compassion, person-centredness, and ethics. A curriculum can reflect different emphases, i.e., knowledge, acting or being [28]. The curriculum is more than a description of a course, lesson plans, and evaluation methods. It reflects what knowledge is being recognised as essential within the profession, but it should also harmonise with the overall program (in this case, the nursing program), and the evaluation methods must fit with the learning objectives. All this must be adapted to the student group, its background, knowledge, and expectations and therefore, the curriculum development should start with the learning objectives [29] or the competencies which the students should have developed in the course. See Table 1 for course competencies.

**Table 1:** Course competencies.

• Use effective communication strategies in different situations. • Explain the therapeutic relationship and its impact on patient care. • Reflect on the impact of one's own communication practices on the formation of therapeutic relationships to patients. • Critically address the basic aspects of health education, such as its purpose, ethics, key learning theories, and the educational process. • Develop and justify a lesson plan for a client on specific topics.

#### 2.2.1 The content

The course content was based on the course competencies, and emphasis was put on students exploring their own attitudes and behaviour in the role of the nurse (being) and gaining skills and competence in communication and the teaching role (acting). Less emphasis was placed on pure theoretical knowledge. The communication part of the course focused on introducing and practicing basic concepts in communication, such as verbal and nonverbal communication, factors that facilitate or hinder the flow of communication, active listening, the role of communication in creating a quality therapeutic relationship, and self-reflection of how one's own values and communication style influence the communication dynamic. The role-playing exercises chosen for practicing communication skills were influenced by motivational interviewing and focused on developing empathic listening skills, such as open-ended questions, affirmations, reflections, and summaries.

For the patient education curriculum, the main emphasis was on introducing the concept and examining its importance through different perspectives such as ethics, self-care, empowerment, and outcomes of healthcare. We focused on the role of the nurse as an educator and discussed what that encompasses. The teaching process was introduced, starting with assessing the learning needs of patients, creating learning objectives, choosing appropriate teaching methods and materials, preparing the education, teaching, evaluating the outcomes with a focus on the teach-back method, and where and how to document patient education.

#### 2.2.2 The teaching methods

The choice of teaching methods and course activities was guided by the literature and higher education principles that emphasize active involvement and the use of experiential learning as the most efficient method of communication training [18].

We chose a mixture of methods that included both individual learning and group work. The methods included role-playing, group discussions, short presentations, scripts, on-line videos, case studies, the Jigsaw puzzle method [30] and the Fishbowl [31] to engage students. At the beginning of the course students were asked to describe their background and experience of communication in healthcare and their expectations of the course and at the end of the course to reflect on those expectations and how they had been met during the course.

The choice of teaching methods was, however, limited by several factors, such as the large group of students (over 80 and up to 125), financial restrictions by the university (limiting the possibilities of teaching in smaller groups), and the available accommodation for teaching (lack of large rooms and suitable set-up for role-playing and group-work).

#### 2.2.3 Student assessment

Various formative assessment methods were incorporated throughout the course instead of a summative final examination. The assessment methods were chosen to reflect the course competencies, focusing on experiential learning. Students were given weekly assignments to ensure continuity of learning and ongoing feedback. These assignments included journals in which students reflected on their developing communication style and how their style was impacted by the course material, personal values, and previous experiences. The students also analysed communication in videos, recorded themselves teaching (role-playing) in the classroom and analysed their performance, and interviewed people who had been recently ill about their educational needs. Assessments also included theoretical essays on topics like health literacy and the professional role of the nurse as an educator. The final assignment was a teaching plan for a patient/client where the student was expected to develop a comprehensive plan that incorporated all course material.

#### 2.2.4 Organisation and environment

The course was developed by three main teachers in collaboration with the university's Director of Educational Development. A team of eight teachers ran the course each year. Class size varied each week, with students meeting in groups of 12, 25, 40, or the entire cohort (80–125) based on that week's teaching and learning modalities. The course ran over 13 weeks and met in person weekly. Mandatory attendance (80–100%) was required in each class. Those who failed to meet this requirement were given the opportunity to complete a written assignment instead. This alternate assignment was later changed to an oral exam. Classes were held in large lecture halls.

## 3 Methods

Several methods were used to evaluate the course, including student feedback on teaching methods in 2018, an online survey in 2023, and written teachers' summaries of their experiences after each semester. Feedback from students is of great value in guiding the development and design of a course [32]. Using self-reports on motivation and skills development has proved useful for gathering data and is highly reliable and helpful in assessing teaching quality [33]. Testing and evaluation were mainly based on two theoretical frameworks or models: The MUSIC® Model of Motivation [22], [23] and self-regulation [19].

### 3.1 Data sources

#### 3.1.1 Student diaries about teaching methods

The first cohort of students (in 2018) was asked to participate in this study by writing and submitting a short diary about their experiences with the weekly teaching methods.

#### 3.1.2 Survey – measures

The 2023 cohort of students (*N* = 117, 3 males) were invited to participate in an online survey exploring their backgrounds, motivation for learning, self-regulation, and perspectives on the teaching methods and course objectives. The online survey was opened on the final day of the course and stayed open for two weeks.

##### 3.1.2.1 Motivation

We used the 19-item version of the MUSIC Model of Motivation Inventory along with the three item Interest Scale from the shorter 18 item version, both translated and validated for use with college students [34], [35]. The 19-item version has four items for eMpowerment (α = 0.84), four items for usefulness (α = 0.88), four items for success (α = 0.90), three items for interest (α = 0.91), and four items for caring (α = 0.88). The three item interest scale from the 18-item version was also used. The items were rated on a six-point Likert scale (1 = strongly disagree to 6 = strongly agree) with higher scores reflecting stronger motivation.

##### 3.1.2.2 Self-regulation in learning

The eight-item self-regulation scale (SRL) from the Student Perceptions of Classroom Knowledge-Building (SPOCK) was used to measure self-regulation [36]. The items were rated on a five-point Likert scale (1 = almost never to 5 = almost always), with higher scores indicating a higher degree of self-regulation.

##### 3.1.2.3 Teaching methods and teaching environment

Four items assessed students' perception of teaching methods, including three items from the longer version of the MUSIC Model of Motivation Inventory [37]. In addition, the students answered 11 questions from the interest scale of the original college version, and on the effectiveness of the different teaching methods. All these questions were rated on the same six-point Likert scale as the motivation scale above.

#### 3.1.3 Teachers' reports and meeting materials

Notes from meetings with the teachers and the Director of Educational Development during the preparation phase of the course and from annual teachers' meetings at the end of each semester (2017–2023) were analysed for this study. The teachers reflected on their experience teaching the students and reading their assignments, which included students´ reflections on their learning.

### 3.2 Data analysis

For quantitative data, descriptive statistics were used (means and standard deviations for continuous variables and frequency (proportion) for categorical variables). SPSS, version 27.0 was used for the data analysis. Mean scores and standard deviations were calculated for each of the MUSIC subscales, each item of the SPOCK self-regulation scale, and students' assessments of teaching methods.

For qualitative data, including students' diaries, teachers' meeting notes and summaries, descriptive content analysis was used [38]. With this approach, codes are derived from theory or relevant research findings and defined before or during data analysis, thus supporting or extending the existing theory [38]. The students‘diaries, which reflected on teaching sessions and learning methods (role-playing, lecture, group-work and discussions), were read thoroughly and then categorized into pros and cons of each method and a summary was written up.

### 3.3 Ethics

The Ethics Committee of the Universities for Scientific Research reviewed and approved the study per Icelandic regulations in 2018 and again in 2023.

## 4 Results

In this section, we combine results from the survey, students' diaries, and teachers' notes structured in accordance with the survey. A total of 70 students participated in the 2023 survey (60% response rate), 64 students returned a total of 304 diary notes, and six teachers provided notes about the preparation of the course and their teaching experience during the period of 2017–2023. The demographics data is presented in Table 2. Most of the participants were employed (*n* = 68, 97.1%), with 85.7% (*n* = 60) of those employed working in a healthcare related field.

**Table 2:** Background of survey participants (N = 70).

Demographic	n (%)
Age	
18–25 yrs	50 (71.4)
26 yrs. and more	20 (28.6)

Employed	
No	2 (2.9)
Yes	68 (97.1)
Within healthcare (yes)	60 (85.7)

Yes	
On and off	12 (17.1)
1–10 h/week	15 (21.4)
11–20	26 (37.1)
21–30	11 (15.7)
31–40	3 (4.3)
>40	1 (1.4)

Marital status	
Married/cohabiting	30 (42.9)
Single	30 (42.9)
Other	10 (14.2)

Children	
One child	5 (7.4)
Two or more children	7 (10.3)
None	56 (82.3)

### 4.1 Motivation

The average scores for the five subscales of the MUSIC Model representing students' motivation ranged from 4.16 to 5.02. See Table 3 for detailed results.

**Table 3:** Mean scores of the MUSIC factors.

MUSIC factor	Mean	SD
eMpowerment	4.45	0.85
Usefulness	4.81	0.85
Success	4.99	0.70
Interest	4.16	1.08
Caring	5.02	5.02

Note. Possible scores 1 (strongly disagree) to 6 (strongly agree), higher scores = stronger motivation.

The teachers noted from conversations with students and written assignments that at the beginning of the course, the students' expectations were low. Many students thought communication and patient education should not require special training and “did not sound very interesting.” At the end of the course, many students from all cohorts reflected on, in their assignments, how their interest in the subject had increased along with their understanding of its complexity and value within nursing and healthcare. However, what seemed to be easy at first turned out to be more complicated, for example, asking open questions or using reflections in conversations. Also, students asked for more training in caring for “difficult patients” and relatives, and patients who have received “bad news”.

### 4.2 Self-regulation

Six of the eight self-regulation statements were rated lower than 4.0. See Table 4 for detailed results.

**Table 4:** Student perceptions of classroom knowledge-building scale (SPOCK).

Question	Mean (SD)
In this class, I try to determine the best approach for studying each assignment.	4.38 (0.76)
In this class, I try to monitor my progress when I study.	4.23 (0.80)
In this class, I focus on understanding the important ideas in what I am reading or studying.	3.95 (0.98)
In this class, I check myself to see how well I am understanding what I am studying.	3.91 (0.78)
In this class, I set goals for myself which I try to accomplish.	3.80 (0.97)
In this class, I make plans for how I will study.	3.74 (1.03)
In this class, I use different ways to organize my thoughts, such as diagrams, charts, timetables, etc.	3.39 (1.18)
In this class, I take notes and jot down questions when I am reading the class materials.	3.14 (1.05)

Note. Possible scores from 1 (almost never) to 5 (almost always), higher scores = higher degree of self-regulation.

### 4.3 Teaching methods, teaching environment, and evaluation

From the survey we learned that the students perceived the teaching to be student-centered (*M* = 4.92, *SD* = 0.89), focused on critical thinking (*M* = 5.17, *SD* = 0.78), and they found the class to be focused more on long-term, usable knowledge rather than purely theoretical (*M* = 4.97, *SD* 0.88).

From both the 2023 survey (see Table 5) and students' diaries (in 2018) we learned that the diverse teaching methods were met with a mixture of enthusiasm and anxiety.

**Table 5:** Student perceptions of the teaching methods.

Teaching method	Mean	SD
Small group discussions	4.92	0.82
Case studies	4.86	0.89
Role-playing	4.52	1.23
Designing patient education	4.37	1.11
Journaling	4.25	1.25
Jigsaw puzzle	4.13	0.95

Note. Possible scores from 1 (strongly disagree) to 6 (strongly agree), higher scores = more effective learning method.

The students appreciated being actively involved in each class, enjoyed small group discussions, and understood the value of roleplaying. At the same time, they felt stressed, awkward, and uncomfortable talking about themselves (e.g. why they chose? nursing, their strengths and weaknesses, their personal values in life, etc.). Students often stated, “*I don't like these exercises, but I know it is good for me”* or “*I am very shy and don't like working in groups, but I know it is good for me to be dragged outside my comfort zone.”* They appreciated being given feedback and role-playing situations that were realistic for the fast hospital environment: “*it was very helpful when the teacher could give us feedback when we were doing the exercises (role-playing)”* and “*the exercise was so good to practice fast thinking and assessing the situation immediately*”. Of note, many students commented that the initial stress and anxiety about participating in the role-playing exercises and discussions would wear off, and in the final weeks, they felt much better.

The students generally preferred smaller student groups of 12 (*M* = 4.62, *SD* = 1.32) or 25 (*M* = 4.63, *SD* = 0.99) over groups of 40 students (*M* = 4.04, *SD* = 1.38). They wanted to be able to work with their friends and other students they already knew (sometimes they were assigned to groups by random or number), but at the same time they would say *“I prefer to work with my friends but I know it is good for me to work with others*” or “*this was a great opportunity to get to know people I have never spoken to before*”.

Both students and teachers struggled with challenges in the classroom setting. The classrooms were large lecture halls, which were not conducive to the teaching methods that emphasised group work, discussions, and role-playing. Having multiple small groups working in one room was noisy and made it difficult to concentrate and engage.

The organisation of the course was also challenging. Students struggled to keep up with the frequency of assignments. The teachers also had difficulty keeping up with grading and providing timely feedback.

## 5 Discussion and conclusion

### 5.1 Discussion

This exploratory case study describes the development of a new course in communication and patient education using student feedback and teachers' experiences over six years. Based on the philosophy and perspectives of nursing education, we used experiential learning strategies in the development of this course to promote critical thinking and self-direction [39]. The feedback from students was essential in guiding the design and development of this course. Based on student's feedback, the content of the course has been gradually adjusted, supported by the European consensus on learning objectives [12] and the new patient education guidelines [6]. For example, we have incorporated more content on difficult situations, communication challenges, and the assessment of educational needs.

We evaluated the motivational context of the course and found acceptable scores for all components, with the lowest scores on the interest scale and the highest on the caring scale. These results are similar to those found by Jones et al. [35] among almost 3000 undergraduate students in the United States, where caring scored the highest and usefulness and success scored lowest, though all above a mean of 4.0, similar to our study. In a study from New Zealand, the scores were a little lower than ours but still between somewhat agree and agree with caring the highest but success the lowest [40], similar to the study mentioned above. In our case, success was high (M = 4.99) indicating that students did believe that they could be successful. We could speculate that the progress made with redesigning the course with clearer directions and possibly more experienced facilitators might have contributed to this result.

In our study the interest factor was the lowest of all the MUSIC factors. A possible cause for the lower score may have been that students were anxious about their performance in the role play. Role play has, at this stage (2nd yr fall semester), not been a common experiential learning strategy in their study environment. Studies have shown that stress and anxiety can reduce learning while positive emotions enhance learning [41], [42]. In simulation studies it is known that a psychologically safe environment needs to be created to foster students´ engagement [42], [43]. The same is likely to be true for the less complicated environment of role play. It is known that facilitators can influence the situation with a warm attitude and open body language [44], [45] and by using tailored scaffolding [46], close supervision and reflective debriefing [47]. Useful frameworks that improve psychological safety and stress during role-play have also been developed [48]. Implementing one of those in this course might reduce anxiety and increase students´ interest in experiential teaching strategies.

The self-regulation scale measures the processes by which students reach their goals. The results show that these students struggled with many aspects of self-regulation, including taking notes, jotting down questions, organizing their thoughts in different ways, and setting goals. The previous classes in the nursing program are primarily science classes, taught with large lectures and a final test as the sole form of assessment. In the communication class, the students were introduced to various new teaching and learning methods, including weekly assignments and ongoing assessments. The students understandably struggled to adapt quickly to all these changes and to keep up with their assignments. It became apparent that students needed to be first taught how to self-reflect, manage their time, and monitor their learning needs. The students also needed to understand the value of experiential learning and ongoing, formative assessment. As a result, the teachers decided to meet with the students as a full cohort three times during the semester to focus on teaching students how to keep themselves organized and to gradually introduce new teaching methods. This laid the foundation for the active learning they would later practice in their small groups. We have found these sessions to be essential in helping the students self-regulate their learning and preparing them for the new learning modalities to come. It also became clear that the experiential methods needed to be woven throughout the nursing curriculum to be most effective.

Through this journey, we have learned several key lessons in motivating students and encouraging self-regulation. Students´ perception of the learning environment is strongly connected to their motivation to participate and engage [23]. The study provided valuable insight into how prepared nursing students were for the variety of teaching methods used in the course. Kember et al. [49] argued that university students often rely on memorization and “surface learning” because they lack the skills needed for a deeper learning. The 2nd year nursing students perceived the course as challenging as it introduced unfamiliar teaching methods and activities that required them to adjust and develop their learning strategies for deeper learning. We found that new teaching methods need to be implemented at a slower pace. This encourages students to understand the purpose of each teaching method and what is asked of them. We also found that smaller groups improved psychological safety for the students. Therefore, we moved to 25-person learning groups, each with their own faculty member, to facilitate the active learning methods.

### 5.2 Innovation

EBP in nursing education supports experiential learning, however, the incoportation of this type of learning into traditional lecture models continues to be a challenge faced by many undergraduate nursing programs. In this paper, we discuss both our struggles and successes, to serve as a potential guide for others attempting this type of curriculum revision. We learned several key lessons about using self-regulation theory to help guide and motivate students through these new experiences. These include introducing new methods one at a time, at a slow pace, to allow students to adapt and get comfortable with them, along with teaching students why these methods are being incorporated and how they encourage deeper learning. Additionally, we learned that working in smaller groups whenever possible provides a better individual experience and a more psychologically safe environment. Finally, caring, encouraging teachers can have a huge impact and make up for other types of challenges, like lack of space to teach.

### 5.3 Limitations

The implementation of an experiential learning curriculum in a large group university setting is a complex, nuanced process. In sharing our challenges and solutions, we hope to support other nursing education programs as they begin incorporating more active learning modalities into their curriculum. However, there are several limitations of our study. This was a single-site study,which may limit the generalizability of the results. The available data varied by year, with the qualitative and quantitative data coming from different years. There were no clear guidelines on the teaching of communication in nursing at the start of this project, therefore they were incorporated later. There was also limited literature on best practices in supporting the development of psychosocial skills such as communication and education.

### 5.4 Conclusion

Nursing schools need to implement diverse evidence-based teaching methods into their curriculum to support the development of different competencies of their nursing students. Teaching methods such as role-play, group discussions, short presentations, scripts, on-line videos, self-reflection journals, and case studies are all evidence-based teaching methods that influence professional growth in different ways. This is particularly important in courses that focus on the more complex interpersonal roles of the nursing profession, such as communication, patient education, and participation in a multidisciplinary team. These teaching methods can help establish psychological safety and create a supportive environment where nursing students feel they can practice these skills without harming patients. It is crucial to incorporate these new methods gradually and to scaffold students' understanding of the active teaching modalities along with their purpose for them to be most effective. When caring educators provide students with such opportunities, nursing schools are able to achieve their purpose, which is to graduate clinically competent nurses that provide safe, high-quality, and compassionate care that meets patient's needs and the complex demands of modern healthcare.

## CRediT authorship contribution statement

**Brynja Ingadóttir:** Writing – review & editing, Writing – original draft, Validation, Supervision, Resources, Project administration, Methodology, Investigation, Funding acquisition, Formal analysis, Data curation, Conceptualization. **Ásta Bryndís Schram:** Writing – review & editing, Writing – original draft, Validation, Methodology, Data curation, Conceptualization. **Helga Sif Friðjónsdóttir:** Writing – review & editing, Writing – original draft, Conceptualization. **Jennifer A. Wentzel:** Writing – review & editing, Writing – original draft, Validation, Software, Methodology, Formal analysis, Conceptualization.

## Declaration of competing interest

All articles must include a separate file containing a statement declaring any competing interests that relate to any authors. The below is the format. Please click either of the two options and submit the form.

The authors declare that they have no known competing financial interests or personal relationships that could have appeared to influence the work reported in this paper.
